# Poor Mental Health in Caregivers of Children with Attention-Deficit/Hyperactivity Disorder and Its Relationships with Caregivers’ Difficulties in Managing the Children’s Behaviors and Worsened Psychological Symptoms during the COVID-19 Pandemic

**DOI:** 10.3390/ijerph18189745

**Published:** 2021-09-16

**Authors:** Hui-Wen Tseng, Ching-Shu Tsai, Yu-Min Chen, Ray C. Hsiao, Fan-Hao Chou, Cheng-Fang Yen

**Affiliations:** 1 School of Nursing, Fooyin University, Kaohsiung 831301, Taiwan; huiwentr@gmail.com; 2 College of Nursing, Kaohsiung Medical University, Kaohsiung 807378, Taiwan; 3 Department of Child and Adolescent Psychiatry, Kaohsiung Chang Gung Memorial Hospital, Kaohsiung 833401, Taiwan; jingshu@cgmh.org.tw; 4 College of Medicine, Chang Gung University, Taoyuan City 33302, Taiwan; 5 Department of Psychiatry, Kaohsiung Medical University Hospital, Kaohsiung 807377, Taiwan; bluepooh79@msn.com; 6 Department of Psychiatry and Behavioral Sciences, University of Washington School of Medicine, Seattle, WA 98195, USA; rhsiao@u.washington.edu; 7 Department of Psychiatry, Children’s Hospital and Regional Medical Center, Seattle, WA 98105, USA; 8 Department of Psychiatry, School of Medicine, College of Medicine, Kaohsiung Medical University, Kaohsiung 807378, Taiwan; 9 College of Professional Studies, National Pingtung University of Science and Technology, Pingtung 91201, Taiwan

**Keywords:** attention-deficit/hyperactivity disorder, caregiver, COVID-19, infectious disease, mental health

## Abstract

The coronavirus disease 2019 (COVID-19) pandemic has thrown out a challenge to caregivers of children with attention-deficit/hyperactivity disorder (ADHD). The present study examined the factors related to the poor general mental health state of the caregivers of children with ADHD during the COVID-19 pandemic, including (1) difficulties of caregivers in asking their child to adopt protective behaviors against COVID-19, (2) difficulties of caregivers in managing the child’s daily performance, and (3) worsened psychological symptoms in children. In total, 161 caregivers completed an online questionnaire to provide data regarding their general mental health state and difficulties in asking their child with ADHD to adopt protective behaviors against COVID-19 and in managing the child’s after-school learning, sleep routine, and internet use, as well as worsened psychological symptoms. The results of multivariate logistic regression analysis demonstrated that caregivers’ difficulties in managing ADHD children’s self-protective behaviors and after-school learning and the children’s worsened emotional symptoms were significantly associated with poor caregiver general mental health state. An intervention that enhances the mental health of caregivers of children with ADHD during the COVID-19 pandemic by addressing their difficulties in managing the children’s behaviors and psychological problems is warranted.

## 1. Introduction

### 1.1. Impact of Coronavirus Disease 2019 on the Mental Health of Caregivers of Children

Coronavirus disease 2019 (COVID-19) has severely affected the lives of people of all ages in myriad ways [1,2,3,4,5,6,7,8]. Caregivers of children face not only the negative influence of the COVID-19 pandemic on their own physical and psychological health and family, social, occupational, and leisure lives, but also the challenges of COVID-19-related adjustment problems among their children [9,10]. For example, a systematic review found that over 50% and 25% of caregivers who were isolated with children developed anxiety and depression, respectively, during the COVID-19 pandemic [11]. Caregiver–child dyadic stress perceived by caregivers exacerbated the behavioral and emotional problems of children during quarantine for COVID-19 [10]. The mental health problems faced by the caregivers may further increase caregiver–child conflicts during the COVID-19 pandemic [12].

### 1.2. Mental Health Problems among Caregivers of Children with Neurodevelopmental Disorders during the COVID-19 Pandemic

Research has found that the COVID-19 pandemic has posed unique challenges for caregivers of children with neurodevelopmental disorders (NDDs), such as autism spectrum disorder (ASD) and attention-deficit/hyperactivity disorder (ADHD). The COVID-19 pandemic not only disrupted the lifestyles and daily routines and deteriorated the behavioral problems in children with NDDs, but also obstructed the social and medical support for caregivers [13]. Caregivers of children with ADHD or ASD reported higher levels of burden, depression, anxiety, and stress [14] and a greater decrease in their quality of life compared with caregivers of children with normal development during the pandemic [15].

No study has yet examined the mental health state of the caregivers of children with ADHD (hereafter referred to as “caregiver mental health”) and their related factors during the COVID-19 pandemic. Several issues related to caregiver mental health warrant further investigation. First, research has found that individuals with ADHD have a significantly increased risk of COVID-19 infection and poor outcomes [16,17,18,19]. Inattention might place individuals with ADHD at an increased risk of forgetting to wear face masks or maintaining social distancing [16,19]. Therefore, caregivers of children with ADHD may have high care burden and, thus, experience mental health problems. However, the relationship between poor mental health and the difficulties in asking the children with ADHD to adopt self-protective behaviors against COVID-19, such as washing hands frequently, keeping the mask on at all times, practicing social distancing, and not touching their mouth and nose among their caregivers, is unclear.

Second, studies before the COVID-19 pandemic have demonstrated that children with ADHD experience greater difficulties in interacting with caregivers [20,21], academic learning [22,23], planning, and time management [24] compared with their peers without ADHD. Studies have further demonstrated that during the COVID-19 pandemic, children with ADHD had fewer daily routines [25,26], more changes in their sleep pattern [27], more disturbances in sleep quality [27], more difficulties in remote learning [25,28], and more digital media use [29,30,31,32] than did those without ADHD. Moreover, caregivers might have less confidence in managing remote learning and more difficulties in supporting the home learning of children with ADHD [25]. It remains undetermined whether the difficulties faced by the caregivers in supporting the learning of children with ADHD, maintaining their sleep routines, and managing their media use are associated with poor caregiver mental health.

Third, emotional dysregulation and oppositional defiant and conduct disorders were commonly presented in children with ADHD before the COVID-19 pandemic [33,34,35]. Research has further found that children with ADHD are more likely than children without ADHD to experience increased symptoms of inattention [26,36], hyperactivity/impulsivity [36], opposition defiance [36], anger [26], and depression [31] during the COVID-19 pandemic. A meta-analysis before the pandemic showed that the presence of oppositionality, externalizing behavior problems, and aggression predicted significantly higher levels of parenting stress [37]. Further studies are needed to determine whether worsened psychological symptoms of children with ADHD are associated with poor caregiver mental health.

### 1.3. Study Aim

The present study examined the factors related to the poor caregiver mental health during the COVID-19 pandemic (outcome variable), including (1) difficulties of caregivers in asking their child to adopt protective behaviors against COVID-19, (2) difficulties of caregivers in managing the child’s daily performance, and (3) worsened psychological symptoms in children with ADHD (predictor variables) by controlling for demographics and medications used in the children for treating ADHD (covariates). We hypothesized that poor caregiver mental health is significantly associated with the difficulties faced by the caregivers in asking their child to adopt self-protective behaviors and in managing the child’s daily performance and worsened psychological symptoms of the children during the COVID-19 pandemic.

## 2. Methods

### 2.1. Participants

This online questionnaire survey study was conducted between 13 October 2020, and 12 May 2021. Three representative associations for caregivers of children with ADHD in Taiwan agreed to post the link to our online questionnaire in their Facebook groups and in LINE (a direct messaging app) for caregivers of children with ADHD. Those who were interested in participating in this study could approach the online survey questionnaire via the link. At the beginning of the online questionnaire, the goal, recruitment criteria, and procedures of the study were explained to potential respondents. Individuals who were caregivers of children with ADHD, those aged ≥20 years, and those living in Taiwan, were included in the study. Caregivers of children with ADHD could press the button “agree to participate” and go to the research questionnaire website or press the button “disagree to participate” and leave the advertisement. The anonymity and confidentiality of the online questionnaire were emphasized when inviting the caregivers of children with ADHD to participate in this study. Regarding the sample size, we used the rule-of-thumb proposed by Green (*N* = 50 + 8 * number of independent variables) [38] to estimate the number of participants needed for linear regression analysis. There were 13 independent variables in this study; therefore, we estimated the number of participants to be 154. In total, 161 caregivers pressed “agree” to participate in this study, and 8 respondents pressed “disagree” and declined to participate. This study was approved by the Institutional Review Board of Kaohsiung Medical University Hospital (KMUHIRB-EXEMPT(I) 20200018). Our study participants were given no incentive for participation.

### 2.2. Measures

#### 2.2.1. General Mental Health State of the Caregivers

The five-item Brief Symptom Rating Scale (BSRS-5) was used to assess the self-reported general mental health state of the caregivers during the week preceding the study [39]. The full scale contained the following five items of psychopathology: (1) feeling tense or keyed up (anxiety); (2) feeling low in mood (depression); (3) feeling easily annoyed or irritated (hostility); (4) feeling inferior to others (interpersonal hypersensitivity: inferiority); and (5) having trouble falling asleep (insomnia). The participants were asked to rate symptoms on a 5-point scale: 0, not at all; 1, a little bit; 2, moderately; 3, quite a bit; and 4, extremely, and a total score was calculated for each participant. The BSRS-5 has been reported to have satisfactory psychometric properties as a measure of psychiatric morbidity in medical settings and in the community [39,40,41]. Its Cronbach’s α value in this study was 0.895. The cutoff of total BSRS-5 score ≥ 6 has been identified by receiver operating characteristic curve analysis to discriminate between the individuals with and without psychiatric diagnoses [39]. Therefore, the participants with total BSRS-5 score ≥ 6 were classified as having a poor caregiver mental health.

#### 2.2.2. Difficulties Faced by Caregivers in Asking Their Child to Adopt Self-Protective Behaviors against COVID-19

Six items were used to determine how often the caregivers experienced difficulties while asking their child to adopt protective behaviors against COVID-19. The first four items assessed caregivers’ difficulties in asking their child to wash hands frequently, keep the mask on at all times, avoid going to crowded places, and practice social distancing recommended by the Centers for Disease Control and Prevention to protect against contracting COVID-19 [42]. We also inquired among the caregivers in outpatient services regarding their experiences in taking care of their children with ADHD during the pandemic and developed another two items to assess their difficulties in asking their child not to touch their mouth and nose as well as objects and other people in public places. Each item was rated on a 4-point scale from 0 (never) to 3 (often). The questions and scoring are listed in Table 1. A total score indicated the levels of difficulties the caregivers faced in asking their child to follow the protective behaviors against COVID-19. The Cronbach’s α value in this study was 0.818. The absolute z-values of skewness and kurtosis were 1.57 and 2.47, respectively. According to Kim [43], the absolute z-values of skewness and kurtosis < 3.29 indicate normal distribution for medium-sized samples (50 < *n* < 300). Accordingly, the variable of the caregivers’ difficulties in asking their child to follow the protective behaviors against COVID-19 was normally distributed.

#### 2.2.3. Caregivers’ Difficulties in Managing Their Child’s Daily Performance

We reviewed the results of previous studies on caregivers’ difficulties in managing ADHD children’s daily performance [25,31]. Accordingly, we developed five items to determine how often the caregivers faced difficulties in managing children’s three dimensions of daily performance during the COVID-19 pandemic, including after-school learning, sleep routine (two items, namely going to bed and waking up on time; Cronbach’s α value: 0.827), and internet use (two items, namely internet use via smartphone and personal computer; Cronbach’s α value: 0.913). Each item was rated on a 4-point scale from 0 (never) to 3 (often). The questions and scoring are listed in Table 1. The scores of the three variables indicated the levels of difficulties faced by the caregivers in managing children’s after-school learning, sleep routine, and internet use. The absolute z-values of skewness and kurtosis of these three variables ranged from 1.03 to 3.14, indicating that they were normally distributed.

#### 2.2.4. Changes in the Psychological Symptoms of the Children

We reviewed the results of previous studies on the psychological symptoms of children with ADHD during the COVID-19 pandemic [26,31] and developed seven questions to examine the changes in their child’s symptoms of inattention, hyperactivity, impulsivity, anger, oppositional defiance, depression, and anxiety before and during the pandemic. Each item was rated as 0 (improved), 1 (no change), 2 (mildly worsened), or 3 (significantly worsened). The questions and scoring are listed in Table 1. Respondents who rated 2 or 3 on any item of inattention, hyperactivity, or impulsivity were classified as having children who had worsened ADHD symptoms during the COVID-19 pandemic. Respondents who rated 2 or 3 on any item of anger or opposition were classified as having children who had worsened opposition/defiance during the COVID-19 pandemic. Respondents who rated 2 or 3 on any item of depression or anxiety were classified as having children who had worsened emotional symptoms during the COVID-19 pandemic.

#### 2.2.5. Medication Use among Children for Treating ADHD

One question derived from the Pediatric Compliance Self-Rating (PCSR) [44] was used to assess the frequency of medication prescribed by the doctors to the child for treating ADHD. The original PCSR is scored on a scale of 1–7 (1 = “never took medication” to 7 = “always took medication”). We simplified the item into a 4-point scale from 0 (never) to 3 (often). The questions and scoring are listed in Table 1. Those who scored 3 were labelled to have children who regularly used medication for ADHD; those who scored less than 3 were labelled to have children who had never or had not regularly used medication for ADHD.

#### 2.2.6. Demographics

Data regarding the sex (0 = male; 1 = female) and age of the caregivers and the sex (0 = boys; 1 = girls) and age of the children were collected.

### 2.3. Statistical Analysis

Data were analyzed using SPSS version 24.0 (SPSS Inc., Chicago, IL, USA). Caregiver mental health, demographics, and difficulties in managing their child’s behaviors and the children’s demographics, medication use, and worsened psychological symptoms were analyzed and are expressed as percentage and mean with standard deviation. The associations between caregiver mental health and the difficulties in asking their child to adopt self-protective behaviors and in managing the child’s after-school learning, sleep, and internet use, and children’s worsened psychological symptoms were examined using multivariate logistic regression analysis, which was adjusted for children’s demographics and medication use. A two-tailed *p* value of <0.05 indicated statistical significance. We used the conditional index to examine the problem with multicollinearity; a value over 30 indicates a serious problem of multicollinearity [45].

## 3. Results

The general mental health state, demographics, and difficulties in managing their child’s behaviors and the demographics, medication use, and worsened psychological symptoms of the children are shown in Table 2. In total, this study included 139 female and 22 male caregivers (mean age: 42.8 ± 5.9 years) and 131 boys and 30 girls with ADHD (mean age: 11.3 ± 3.8 years); 113 (70.2%) children were on regular medication for treating ADHD. A total of 37 (23%) caregivers had a poor general mental health state.

**Figure 1:**
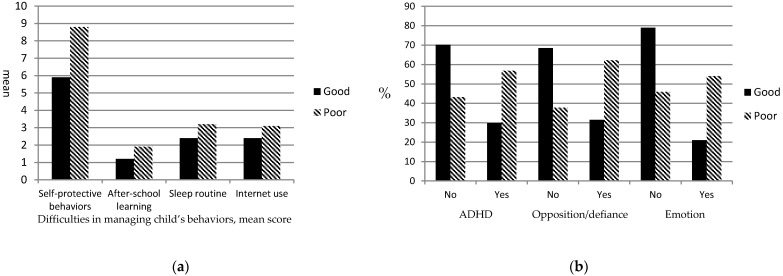
(**a**) Difficulties in Managing Child’s Behavior and (**b**) Worsened Psychological Symptoms between Caregivers with Good and Poor Mental Health.

The results of Chi-square and *t*-tests of demographics, difficulties in managing children’s behaviors, and worsened psychological symptoms between the caregivers with good and poor mental health are shown in Table 3. The results indicated that, compared with the caregivers with good mental health, the caregivers with poor mental health were more likely to be female, experienced greater difficulties in asking their child to adopt self-protective behaviors against COVID-19, experienced greater difficulties in managing the child’s after-school learning and sleep routine, and had a higher chance of having a child with worsened ADHD, opposition/defiance, and emotional symptoms during the COVID-19 pandemic (Figure 1). The caregivers with poor mental health also tended to experience greater difficulties in managing internet use among children with ADHD than did the caregivers with good mental health. No significant difference was observed in the caregivers’ age and children’s demographics and medication use for ADHD between the caregivers with good and poor mental health.

The results of multivariate logistic analysis of the factors related to caregiver mental health are shown in Table 4. The results of Model 1 indicated that, after controlling for demographics and medication use, difficulty in asking their child to adopt self-protective behaviors against COVID-19 was significantly associated with caregiver mental health (odds ratio (OR) = 1.152, 95% confidence interval (CI): 1.054–1.259). The difficulties experienced by the caregivers in managing the daily performance of the children were included in Model 2, and the results indicated that, after controlling for demographics and medication use, the difficulty in managing the child’s after-school learning was significantly associated with caregiver mental health (OR = 1.368, CI: 1.133–1.652). The worsened psychological symptoms of the children were included in Model 3, and the results indicated that, after controlling for demographics and medication use, worsened emotional symptoms of the children was significantly associated with caregiver mental health (OR = 1.360, CI: 1.103–1.677). The conditional index was 27.248 in Model 1, 29.294 in Model 2, and 28.559 in Model 3.

## 4. Discussion

In the present study, we found that 23% of caregivers of children with ADHD had a poor mental health state. The difficulties faced by the caregivers in asking their child to adopt self-protective behaviors and in managing the child’s after-school learning and the worsened emotional symptoms of the children during the COVID-19 pandemic were significantly associated with poor caregiver mental health.

### 4.1. Difficulties in Managing Children’s Behaviors and Poor Caregiver Mental Health

The context of the interaction between caregivers and their children with ADHD during the COVID-19 pandemic is different from the context before the pandemic. Since the emergence of the COVID-19 pandemic, the Taiwanese government has implemented a program to reduce the risk of spreading of COVID-19, including asking people to wear a mask when out of the home, mandating social distancing, and encouraging people to wash hands frequently. A previous study found that most adult people in Taiwan had no difficulty in adopting self-protective behaviors against COVID-19 [46]. However, according to the Theory of Planned Behavior [47], attitude, subjective norms, and perceived behavioral control all shape an individual’s intention to adopt self-protective behaviors against COVID-19. Moreover, these self-protective behaviors against COVID-19 are different from daily routines; some of them, such as social distancing, even violate the developmental needs of children. Therefore, it is difficult for children to adopt and maintain self-protective behaviors against COVID-19. The core symptoms of ADHD may further increase the risks of forgetting to maintain self-protective behaviors in children with ADHD. Caregivers may have to take huge efforts to explain the necessity of self-protective behaviors, as well as persuade and remind their child with ADHD to adopt them. Caregivers may also worry about the risk of their child contracting COVID-19 if their child shows no or low cooperation with the requirements during the COVID-19 pandemic. Moreover, the public may strongly ask everyone to follow the rule of prevention during the COVID-19 pandemic; caregivers may feel stressed if their child did not adopt and maintain self-protective behaviors. All these predicaments may contribute to the deterioration of caregiver mental health.

The present study found that difficulties in managing the child’s after-school learning were significantly associated with caregiver mental health. The results indicated that, even during the COVID-19 pandemic, managing the learning behaviors of children with ADHD was challenging for their caregivers. The learning behaviors of these children during the pandemic may be influenced by their academic difficulties [22,23], low academic motivation [48], and poorer planning and time management skills [24] originally before the pandemic, as well as by the difficulties in remote learning [25,28] during the pandemic. Other changes that occurred during the pandemic, such as reduced daily routines [25,26], sleep pattern [27], and emotional well-being [26,31,36,37], may have also indirectly contributed to the learning difficulties faced by children with ADHD. Caregivers may face the expectation from school teachers and other family members to supervise their child’s completing homework; the difficulty in managing the child’s after-school learning may threaten caregiver mental health.

In addition to the possibility that the difficulties in managing children’s self-protective and after-school learning behaviors may worsen caregivers’ mental health, it is also possible that poor mental health may compromise caregivers’ cognitive function, managing skills, and patience, which are essential for successfully communicating with children with ADHD and increasing their intention to cooperate with caregivers. Poor mental health and the difficulties in managing children’s behaviors may form a vicious circle. Active interventions are needed for the caregivers of children with ADHD to improve their mental health and abilities to successfully manage their child’s behaviors.

Another possibility that should be noted is that both poor mental health and the difficulties in managing children’s behaviors may result from the third factor. For example, deficiencies of family support and economic embarrassment may increase caregivers’ psychological burden and children’s uncooperativeness with caregivers’ instructions simultaneously. The possible factors accounting for the association between caregivers’ poor mental health and difficulties in managing children’s behaviors should be evaluated.

The present study found that the caregivers with a poor general mental health state tended to experience greater difficulties in managing the child’s sleep routine and internet use than did the caregivers with a good mental health state in the *t*-test. The high prevalence of sleep disturbance [49] and problematic internet use [50,51] in children with ADHD has been well established in previous studies. The lockdown during the COVID-19 pandemic further strengthened the maladaptive sleep patterns and impacted sleep–wake rhythms in children with ADHD [27]. Sleep problems can further worsen the severity of ADHD symptoms [52]. The ADHD children with problematic internet use suffered from more severe ADHD symptoms, negative emotions, executive function deficits, damage on the family environment, pressure from life events, and a lower motivation to learn during the COVID-19 pandemic [53]. Although the associations between caregiver mental health and difficulties in managing children’s sleep pattern and internet use became non-significant in multivariate logistic analysis in this study, caregivers’ difficulties in managing ADHD children’s sleep and internet use behaviors need assessment and intervention by physicians.

### 4.2. Worsened Psychological Symptoms of Children with ADHD and Poor Caregiver Mental Health

Most previous studies have reported that ADHD, opposition defiance, and emotional symptoms worsened in children with ADHD during the COVID-19 pandemic [6,31]. Although a follow-up study reported that these symptoms, except for inattention, in children with ADHD might decrease progressively during the COVID-19 pandemic [36], worsened psychological symptoms might negatively affect the well-being of the children and their caregivers and caregiver–child interactions. The present study demonstrated that worsened emotional symptoms of children with ADHD during the COVID-19 pandemic were significantly associated with caregiver mental health. Children with ADHD may suffer from social isolation, difficulties engaging in online learning, boredom, and worrying about being contracted during the COVID-19 pandemic [28], and therefore suffer from emotional problems; caregivers may experience burden aggravation, followed by the deterioration of mental health. It is also possible that poor mental health worsens the family atmosphere or the caregiver–child communication and then negatively impacts children’s emotions. Caregiver mental health and children’s emotional problems may also be resulted from the common genetic disposition or shared stressors such as economic hardship or domestic violence. The present study indicated that worsened emotional symptoms in children with ADHD during the COVID-19 pandemic need to be actively evaluated and intervened.

### 4.3. Implications

The results of this study indicated that mental health of caregivers of children with ADHD should be emphasized. Intervention programs should be developed in multiple aspects, including caregivers, children with ADHD, health service provision, and health policies. Regarding caregivers, caregivers’ mental health should be routinely assessed to early detect mental health problems. Timely providing psychological and pharmacological assistance may contribute to the improvement of mental health in caregivers. Enhancing caregivers’ skills of managing the behaviors and psychological symptoms of children with ADHD during the COVID-19 pandemic may not only prevent caregivers from helplessness and frustration, but also improve caregiver–child interactions [54]. Shorey et al. recommended a list of strategies for caregivers of children with NDDs during the pandemic, such as scheduling regular online consultations, maintaining online therapy, educating children on the nature of and self-protective behaviors against COVID-19, creating a structured daily schedule and reinforcement system, and selecting child-appropriate activities [13].

Regarding children with ADHD, given that pharmacological treatment significantly reduces the risk of negative outcomes in individuals with ADHD [55], continuing effective pharmacological treatment for ADHD is recommended [45]. In addition, children may suffer from not only ADHD symptoms and related functional deficits but also the impacts of the COVID-19 pandemic. Behavioral interventions should be implemented to improve the mental well-being of children with ADHD and enhance their adjustment to the changes caused by the pandemic [54].

Regarding health service provision, according to the special situations and needs encountered during the COVID-19 pandemic, modified models of health service provision should be developed. For example, intervention programs using telepsychiatry or telepsychology may provide feasible and convenient behavioral interventions for caregivers of children with ADHD [56]. Moreover, case management and active following of those who lost visiting outpatient units may provide timely assistance for the caregivers and their children with ADHD. Healthcare providers should also evaluate the possible mechanisms accounting for the association between caregivers’ poor mental health and difficulties in managing children’s behaviors.

Regarding health policies, based on the ecological system theory [57], macrosystemic factors such as social and cultural factors may influence the mental health and behaviors of caregivers and their children with ADHD. The governments should develop socially and culturally sensitive and specific health policies for their people. For example, vaccination has been expected to stop the spread of COVID-19 [58]. The Centers for Disease Control and Prevention in the United States has approved the Pfizer–BioNTech COVID-19 vaccine for use in teens aged ≥12 years to protect them against COVID-19 [59]. The European Medicines Agency in the European Union has also approved the Pfizer–BioNTech and Moderna COVID-19 vaccines for use in teens aged ≥12 years [60,61]. However, caregiver hesitancy to vaccinate their children against COVID-19 is prevalent [62,63]. Intervention to enhance the intention of caregivers to vaccinate their children with ADHD should take social and cultural factors into consideration.

### 4.4. Limitations

This study has several limitations. First, the participants were enrolled through an online advertisement delivered to caregivers of children with ADHD who joined the associations for such caregivers in Taiwan. Although this was a practical method to recruit participants during the COVID-19 pandemic, this enrollment method may limit representative participants. Second, the cross-sectional research design of our study limited our ability to draw conclusions regarding the temporal relationships of the difficulties faced by the caregivers in managing their child’s behaviors and the worsened psychological symptoms of the children with caregiver mental health. Third, the caregivers provided all the data in this study, likely leading to the problem of shared-method variance resulting from a sole information source, which requires careful consideration. Fourth, this study did not recruit the caregivers of children without ADHD for comparison. Further studies are necessary to examine whether the caregivers of children with ADHD have a worse mental health state and whether the factors related to their mental health state found in this study relate to those observed in caregivers of children without ADHD. Moreover, the sample size was small (*N* = 161); further study on a larger sample of caregivers of children with ADHD is warranted.

## 5. Conclusions

The findings of this study showed that difficulties faced by caregivers of children with ADHD in managing self-protective behaviors against COVID-19 and after-school learning and the children’s worsened psychological symptoms during the COVID-19 pandemic were significantly associated with caregiver mental health. Intervention from the aspects of caregivers, children with ADHD, medical service provision, and health policies should be proposed to enhance caregiver mental health, caregivers’ skills of managing children’s protective and after-school learning behaviors, and children’s psychological well-being during the COVID-19 pandemic.

## Figures and Tables

**Table 1:** Contents of the Research Questionnaire.

Measures	Items		Response Scale
Caregivers’ difficulties in asking their child to adopt self-protective behaviors against COVID-19	How often do you find it difficult to ask your child to do the following things during the COVID-19 pandemic?		0 = never, 1 = seldom, 2 = sometimes, 3 = often
	Item 1: Washing hands frequently		
	Item 2: Keeping the mask on at all times		
	Item 3: Not touching the mouth and/or nose		
	Item 4: Not touching anything in public places		
	Item 5: Not going to crowded places		
	Item 6: Practicing social distancing and not touching others		
Caregivers’ difficulties in managing their child’s daily performance	How often do you find it difficult to manage the following day-to-day activities of your children during school closures and delayed school openings due to the COVID-19 pandemic?		0 = never, 1 = seldom, 2 = sometimes, 3 = often
	After-school learning	Item 1: Asking my child to finish homework	
	Sleep routine	Item 1: Asking my child to go to bed on time	
		Item 2: Asking my child to wake up on time	
	Internet use	Item 1: Managing my child’s internet use via smartphone phones	
		Item 2: Managing my child’s internet use via personal computers	
Changes in psychological symptoms	Compared with those before the pandemic, do the psychological symptoms change during the COVID-19 pandemic?		0 = improved, 1 = no change, 2 = mildly worsened, 3 = significantly worsened
	Item 1: Not being able to concentrate		
	Item 2: Being overactive physically		
	Item 3: Not being able to control their impulsive behavior		
	Item 4: Being easily angered and upset		
	Item 5: Having a tendency to argue and rebel		
	Item 6: Feeling depressed and down		
	Item 7: Feeling anxious and getting worried easily		
Child’s taking medication for treating ADHD	How often does your child take medication prescribed by doctors for treating ADHD?		0 = never, 1 = seldom, 2 = sometimes, 3 = often

ADHD: attention-deficit/hyperactivity disorder; COVID-19: coronavirus disease 2019.

**Table 2:** Demographics, caregiver mental health, and Difficulties in Managing Child’s Behaviors, and Children’s Treatment and Worsened Psychological Symptoms (*n* = 161).

Variables	*n* (%)	Mean (SD)	Range
*Caregivers*			
General mental health state			
Good	124 (77)		
Poor	37 (23)		
Gender			
Male	22 (13.7)		
Female	139 (86.3)		
Age (years)		42.8 (5.9)	22–71
Difficulties in managing child’s behaviors			
Self-protective behaviors against COVID-19		6.5 (4.6)	0–18
After-school learning		1.4 (1.1)	0–3
Sleep routine		2.6 (1.9)	0–6
Internet use		2.6 (2.0)	0–6
*Children*			
Gender			
Boys	131 (81.4)		
Girls	30 (18.6)		
Age (years)		11.3 (3.8)	5–18
Taking medication for ADHD			
No or irregular	48 (29.8)		
Regular	113 (70.2)		
Worsened psychological symptoms			
ADHD			
No	103 (64)		
Yes	58 (36)		
Oppostion/defiance			
No	99 (61.5)		
Yes	62 (38.5)		
Emotion			
No	115 (71.4)		
Yes	46 (28.6)		

ADHD: attention-deficit/hyperactivity disorder; COVID-19: coronavirus disease 2019.

**Table 3:** Comparisons of Caregivers’ Demographics and Difficulties in Managing Child’s Behaviors and Children’s Demographics, Treatment, and Worsened Psychological Symptoms Between Caregivers with Good and Poor Mental Health.

Variables	Caregiver Mental Health		χ^2^ or *t*	*p*
	Good (*n* = 124)	Poor(*n* = 37)		
*Caregivers*				
Gender, *n* (%)				
Male	21 (16.9)	1 (2.7)	4.893	0.027
Female	103 (83.1)	36 (97.3)		
Age (years), mean (SD)	42.9 (6.1)	42.2 (5.4)	0.619	0.537
Difficulties in managing child’s behaviors, mean (SD)				
Self-protective behaviors	5.9 (4.5)	8.8 (4.2)	−3.514	0.001
After-school learning	1.2 (1.1)	1.9 (1.2)	−3.232	0.001
Sleep routine	2.4 (1.9)	3.2 (2.0)	−1.992	0.048
Internet use	2.4 (2.0)	3.1 (2.2)	−1.901	0.059
*Children*				
Gender, *n* (%)				
Boys	100 (80.6)	31 (83.8)	0.185	0.667
Girls	24 (19.4)	6 (16.2)		
Age (years), mean (SD)	11.3 (3.7)	11.2 (4.2)	0.112	0.911
Taking medication for ADHD, *n* (%)				
No or irregular	37 (29.8)	11 (29.7)	0.000	0.990
Regular	87 (70.2)	26 (70.3)		
Worsened psychological symptoms, *n* (%)				
ADHD				
No	87 (70.2)	16 (43.2)	8.959	0.003
Yes	37 (29.8)	21 (56.8)		
Oppostion/defiance				
No	85 (68.5)	14 (37.8)	11.350	0.001
Yes	39 (31.5)	23 (62.2)		
Emotion				
No	98 (79.0)	17 (45.9)	15.286	<0.001
Yes	26 (21.0)	20 (54.1)		

ADHD: attention-deficit/hyperactivity disorder; SD: standard deviation.

**Table 4:** Factors Related to Caregiver Mental Health: Multivariate Logistic Analysis.

Variables	Caregivers’ Poor General Mental Health State		
	Model 1	Model 2	Model 3
	OR (95% CI)	OR (95% CI)	OR (95% CI)
Female caregivers ^a^	6.018 (0.762–47.552)	5.424 (0.685–42.938)	7.433 (0.882–62.662)
Caregivers’ age	0.987 (0.908–1.074)	0.968 (0.895–1.046)	0.992 (0.917–1.072)
Girl child ^b^	0.658 (0.232–1.866)	0.814 (0.294–2.249)	0.793 (0.272–2.312)
Child’s age	1.050 (0.928–1.187)	1.017 (0.900–1.150)	0.967 (0.849–1.101)
Child’s regularly taking medication for ADHD ^c^	0.803 (0.341–1.889)	0.823 (0.348–1.946)	0.976 (0.399–2.384)
Caregivers’ difficulty in asking their child to adopt self-protective behaviors	1.152 (1.054–1.259) **		
Caregivers’ difficulty in managing their child’s after-school learning		1.534 (1.006–2.340) *	
Caregivers’ difficulty in managing their child’s sleep routine		1.050 (0.831–1.327)	
Caregivers’ difficulty in managing their child’s smartphone and internet use		1.037 (0.817–1.317)	
Worsened ADHD symptoms			1.350 (0.501–3.639)
Worsened opposition/defiance			2.204 (0.819–5.932)
Worsened emotional symptoms			2.999 (1.224–7.350) *

ADHD: attention-deficit/hyperactivity disorder; CI: confidence interval; OR: odds ratio. ^a^ Male caregivers as the reference; ^b^ boys as the reference; ^c^ Not taking or irregularly taking medication as the reference. * *p* < 0.05, ** *p* < 0.01.

## Data Availability

The data will be available upon reasonable request to the corresponding authors.
